# Exploring the Capabilities of a Piezoresistive Graphene-Loaded Waterborne Paint for Discrete Strain and Spatial Sensing

**DOI:** 10.3390/s22114241

**Published:** 2022-06-02

**Authors:** Alessio Tamburrano, Alessandro Proietti, Marco Fortunato, Nicola Pesce, Maria Sabrina Sarto

**Affiliations:** 1 Department of Astronautical, Electrical and Energy Engineering (DIAEE), Sapienza University of Rome, 00184 Rome, Italy; marco.fortunato@uniroma1.it (M.F.); nicola.pesce@uniroma1.it (N.P.); mariasabrina.sarto@uniroma1.it (M.S.S.); 2 Nanotechnology Research Center Applied to Engineering (CNIS), Sapienza University of Rome, 00185 Rome, Italy; 3 ICAPGROUP, 04100 Latina, Italy; alessandro.proietti@icapgroup.it

**Keywords:** paint, piezoresistivity, piezoresistive sensor, graphene, tomography, strain, damage detection, object localization

## Abstract

The development of a piezoresistive coating produced from dispersing graphene nanoplatelets (GNPs) inside a commercial water-based polyurethane paint is presented. The feasibility of its exploitation for realizing highly sensitive discrete strain sensors and to measure spatial strain distribution using linear and two-dimensional depositions was investigated. Firstly, the production process was optimized to achieve the best electromechanical response. The obtained materials were then subjected to different characterizations for structural and functional investigations. Morphological analyses showed a homogenous dispersion of GNPs within the host matrix and an average thickness of about 75 µm of the obtained nanostructured films. By several adhesion tests, it was demonstrated that the presence of the nanostructures inside the paint film lowered the adhesion strength by only 20% in respect to neat paint. Through electrical tests, the percolation curve of the nanomaterial was acquired, showing an effective electrical conductivity ranging from about 10^−4^ S/m to 3.5 S/m in relation to the different amounts of filler dispersed in the neat paint: in particular, samples with weight fractions of 2, 2.5, 3, 3.5, 4, 5 and 6 wt% of GNPs were produced and characterized. Next, the sensitivity to flexural strain of small piezoresistive sensors deposited by a spray-coating technique on a fiberglass-reinforced epoxy laminate beam was measured: a high gauge factor of 33 was obtained at a maximum strain of 1%. Thus, the sensitivity curve of the piezoresistive material was successively adopted to predict the strain along a multicontact painted strip on the same beam. Finally, for a painted laminate plate subjected to a mechanical flexural load, we demonstrated, through an electrical resistance tomography technique, the feasibility to map the electrical conductivity variations, which are strictly related to the induced strain/stress field. As a further example, we also showed the possibility of using the coating to detect the presence of conducting objects and damage.

## 1. Introduction

In the last few years, there has been an increasing interest in developing non-destructive techniques to be successfully used for strain and damage detection, as well as new high-performance sensing materials for structural health monitoring (SHM) applications [1,2].

Novel multifunctional materials are often made by adding conductive nanostructures (e.g., carbon nanotubes, carbon nanofibers, multilayer graphenes, etc.) to a polymeric matrix until a percolating network is achieved. Thus, any mechanical stress results in the disruption of or an increase in the conductive channels, with a consequent change in the material’s resistivity. This phenomenon, known as the piezoresistive effect, has been exploited in several research studies. For example, in [3], the authors investigated the strain-dependent electrical resistance of polyvinyl ester-based composites filled with graphene nanoplatelets (GNPs). They produced the nanostructure through a liquid phase exfoliation procedure and subsequently mixed them with the liquid pre-polymer. It was demonstrated that this material has a stable and high piezoresistive response. Using the same technique, Jan and his co-workers [4] produced a thermoplastic polyurethane/graphene nanosheet composite and used it as a sensing layer to measure the deformation of a glass-fiber-reinforced polymer (GFRP) sample. Their sensor showed a linear response, a sensitivity comparable with that of a commercial foil strain gauge, and an unignorable drift during cyclic loading and unloading. As in the previous examples, most of the solutions require the use of either a cyanoacrylate glue or an epoxy-based paste to attach the sensing material to the monitored structure. Unfortunately, the adhesives’ viscoelasticity can negatively affect the sensor output [5]. An attempt to overcome this limitation was proposed in [6], where the authors cast the uncured epoxy/GNP mixture over a carbon fiber/epoxy composite (CFRC) panel. This technique allowed them to directly deposit the sensing material over a structure, but seems infeasible on an industrial scale. Other research groups have adopted wet, non-contacting methods [7,8,9,10,11,12]. In particular, in [10], we developed a GNP-based piezoresistive coating that was able to measure extremely low deformations and detect ultrasonic waves on a CFRC plate. Despite their sensing capabilities, the sensors are based on a binder-free coating of the nanostructures, and they need to be protected somehow from the external environment, which can severely alter their electrical properties in the long run. In [11], we patented a production strategy allowing us to incorporate GNPs in waterborne paints and realize coatings with multifunctional properties. In particular, in [12], we focused our attention on the piezoresistive response of a water-based polyurethane (PU) paint filled with GNPs and optimized it after rheological analyses. Electromechanical tests demonstrated that the sensors could follow the substrate deformation up to 1% with a maximum GF of ~17, and that the adoption of a covering agent helped to mitigate the effects of humidity.

As regards techniques for SHM, guided-waves-based methods have proven to be excellent for identifying strain fields and damage in simple structural components. However, when the structure is particularly complex, the computational effort increases and the method’s accuracy decreases. In addition, a computationally intensive algorithm and a huge network of sensor/actuator are generally needed to measure high spatial strain distributions and detect small amounts of damage [13]. Recently, the combined use of novel sensing skins and electrical tomography (ET) has also gained significant attention for different SHM application scenarios. ET is a non-intrusive technique enabling the mapping of the spatial representation of the electrical conductivity of the monitored object using the measured voltage on its boundaries while passing a current through the object [14]. Electrical resistance tomography (ERT) and electrical impedance tomography (EIT) are the most common implementations of ET. As an example, in [15], the authors developed an ERT system for measuring the voltage at multiple locations of the sensing layer of an epoxy/glass fiber composite with a 0.5 wt% carbon nanotube loading, and they demonstrated that it was able to locate a defect smaller than 0.1% of the total monitored area. A similar work, but using carbon black as the nanofiller, was presented in [16]. Furthermore, in [17], the authors explored the potential of extending EIT to complex shapes for damage detection, such as carbon black-modified glass fiber/epoxy composite tubes, showing that multiple through-holes as small as 7.94 mm can accurately be detected on a tube with an aspect ratio of 2:1. Loyola and co-authors [18] used a spray-coated MWNT-PVDF film and an algorithm developed in-house to perform spatially distributed sensing, obtaining a low reconstruction time and a gauge factor of -0.481. Lestari and co-workers, using the same sensing skin of [18], obtained a maximum gauge factor of 6.225 and demonstrated the ability of the coating to sense the damage mode of unidirectional glass and carbon fiber composites. In [19], non-woven aramid fabric, coated with nanotubes and subsequently infused with epoxy resin, was investigated as a sensing skin. The authors demonstrated the feasibility of locating damage, also providing information on the damage severity, with some overestimation of the size of the damage and imperfect representation of its shape. In [20], the authors showed how the spatial resolution of the ERT can be improved through the use of a Gaussian anisotropic smoothing filter, revealing cuts over a carbon fiber-reinforced polymer composite. In [21], Tallman and others calculated the strains and stresses from conductivity data measured on a carbon nanofiber/polyurethane sensing skin by using a proprietary EIT code, and ANSYS was used to validate the accuracy of the piezoresistive behavior. More recently, the use of artificial intelligence is gaining ground in the identification and localization of structural damage [22,23]. The main advantages are the acceleration of the computation time, the fast acquisition of the input data and the flexibility of the machine learning algorithm to solve high-dimensional and non-linear functions such as the EIT equations.

The main scope of the present study was to characterize the piezoresistive performance of a novel aeronautic-grade waterborne PU paint properly modified by the use of electrically conducting cost-effective GNPs, investigating not only its use for measuring strain at discrete points on structures, as with commercial foil strain gauges, but also the feasibility of realizing a sensing skin that is able to evaluate the strain field over large surfaces. With this intent, we firstly spray-deposited the GNP/PU paint on a FR4 (a flame retardant glass-fiber reinforced resin laminate) beam with an airbrush mounted on a CNC 2D plotter, obtaining a small rectangular-shaped resistive sensor whose sensitivity to strain was assessed through electromechanical tests. Secondly, the paint was sprayed to obtain a piezoresistive strip a few centimeters long on the beam sample, properly contacted with copper pads and wires at eight equidistant points along its length. Thus, during a three-point flexural mechanical test, the variations in the resistance of the seven strip segments between adjacent contacts, constituting a 1D sensor array, were measured, and the recorded values were used to extract the strain along the strip. Next, we implemented an electrical resistance tomography (ERT) setup with the piezoresistive paint used as a 2D sensing layer for electrical conductivity and strain mapping. Additionally, we also showed the possibility of using the coating to identify the position of an object or the presence of damage over the coated surface. To the best of our knowledge, this is the first attempt to produce a skin with a commercial aeronautic grade paint properly modified with cost-effective nanostructures such as GNPs, showing potential application in the field of strain detection in reinforced composite materials.

In particular, Section 2 is focused on a description of the process to realize different piezoresistive samples and on the setups and tests used for the characterizations. Section 3.1 reports the results of the morphological analysis, the adhesion properties of loaded and unloaded PU paint have been addressed in Section 3.2, the electrical and the piezoresistive behavior of the nanostructured coatings is presented in Section 3.3 and Section 3.4 respectively, the piezoresistive response of a linear sensor array is then described in Section 3.5. The last section is dedicated to the characterization of a sensing surface.

## 2. Materials and Methods

### 2.1. Polyurethane/GNP Piezoresistive Paint Production

The conductive paint was produced by adding GNPs to a commercial water-based polyurethane paint with different filler weight percentages (wt%). In particular, weight concentrations of GNPs with respect to the paint ranging from 2 wt% up to 6 wt% were considered.

GNPs were produced by liquid phase exfoliation of thermally expanded graphite intercalation compounds (GIC) provided by Graftech Inc. (Parma, OH, USA) as described in [10].Dried GNPs were added to the PU paint diluted with tap water in the ratio of 10:2 (10 parts by weight of paint to 2 parts by weight of deionized water [12]). The mixture was first homogenized using a high shear mixer and then ultrasonicated for a few minutes to break up micro-agglomerates and to promote the optimum dispersion of GNPs into the paint.

Subsequently, after adding the curing agent (at a 25:100 ratio of crosslinker to polymeric paint) the GNP-based polyurethane paint was spray-deposited on the desired planar substrates through an airbrush mounted on a CNC 2D plotter.

Different mask shapes were applied to the substrate to obtain a single sensor, a strip with multiple sensors, or square and rectangular surfaces for ERT. Notice that, depending on the test typology, either polyethylene terephthalate (PET) sheets or FR4 were used as the substrates to produce specimens. In fact, as will be detailed later, we analyzed the morphology of the coatings, the effect of filler content on the paint’s adhesion to the substrate, and their electrical conductivity and electromechanical response. For the first three types of test, several samples were prepared on flexible and easily cut PET sheets; the piezoresistive response of the loaded PU paint under an applied flexural stress were investigated using FR4 as the substrate.

**Figure 1:**
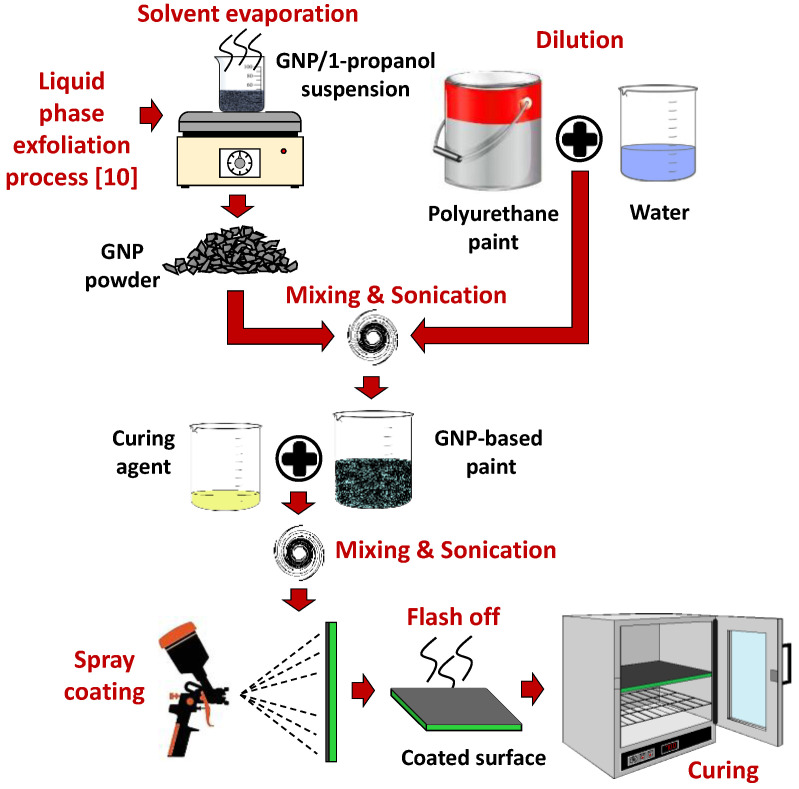
Sketch of the GNP–PU paint production steps.

The wet paint was then kept at room temperature for 10 min before baking to ensure that fast-evaporating solvents left the coating film. Finally, the paint was cured in oven at 70 °C for 1 h. The process is sketched in Figure 1.

### 2.2. Microscopy

The morphology of the coatings was observed through a field emission scanning electron microscope (FE-SEM) (Zeiss Auriga). The fracture surfaces of the films were realized through immersion in liquid nitrogen and sputter-coated with a 10–20 nm Cr layer by a Quorum Tech Q150T sputter coater. For reference purposes, we also investigated the morphology of the neat PU paint.

### 2.3. Adhesion Test

**Figure 2:**
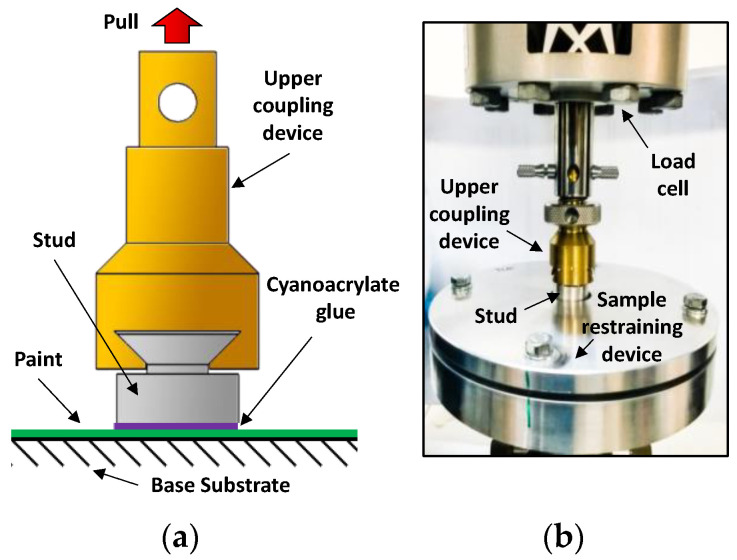
Sketch (**a**) and picture (**b**) of pull-off setup according to ASTM 5179.

One of the most important properties of a multifunctional coating is to adhere to the underlying substrate under specific operating conditions for an expected service life. Therefore, an evaluation of the adhesion of a new paint is fundamental for the assessment of its suitability for the intended purpose. According to the ASTM 5179 standard [24], we investigated the coating–substrate adhesion of the PU-based paint loaded with different GNP concentrations through the use of an universal testing machine (Instron 3366) equipped with a 500 N load cell and a pull-off fixture (Figure 2). Basically, an aluminum stud was first bonded with a cyanoacrylate adhesive to a paint-coated panel (the specimen). Next, the specimen was placed in the restraining device and subjected to a tensile test with an upper coupling adaptor connected to the load cell (upper side) and fitted to the stud (bottom side).

### 2.4. Electrical Properties of the GNP-Filled Paint

The GNP-filled PU paint is basically a two-phase composite whose insulator-to-conductor transition is described by the percolation curve *σ*-*θ* of the mixed polymeric material, where *σ* (S/m) is the effective DC electrical conductivity of the produced samples and *θ* is the GNP weight fraction. In general, the percolation threshold depends on many factors, such as the process, the aspect ratio, the inherent electrical conductivity and dispersion degree of the filler, the polymer type and its interaction with the inclusion.

**Figure 3:**
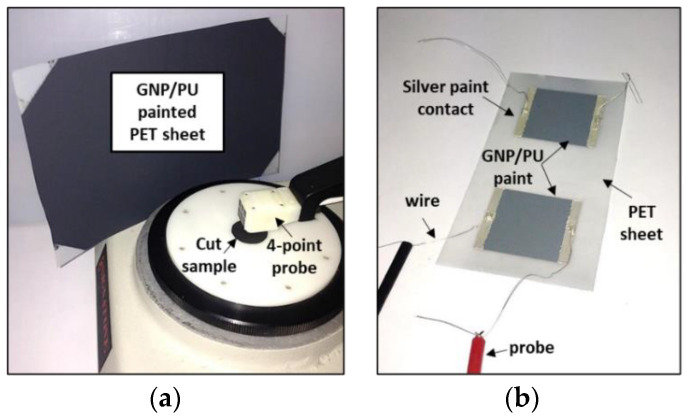
(**a**) Four-point probe setup for measuring film resistance; (**b**) nanocomposites samples with 2 and 2.5 wt% of GNPs.

The sheet resistance *R_s_* of the most conductive film samples, with a weight percentage of GNP equal to 3, 3.5, 4, 5 and 6 wt%, was measured at room temperature through the 4-point probe method (ASTM F390–398) (Figure 3a). The test setup comprised a probe head with four equally spaced collinear tungsten carbide tips connected to an AC/DC current source (Keithley 6221) and a nanovoltmeter (Keithley 2182a). The effective DC electrical conductivity σ of the coatings was then obtained as the inverse of the product between the measured *R_s_* and the thickness of samples.

The other nanocomposites with 2 and 2.5 wt% of GNPs, due to their higher electrical resistance, were characterized with a two-wire volt-amperometric method using a high-resistance meter/electrometer (Keithley 6517B). For this purpose, before measurement, rectangular (4 cm × 3 cm) specimens were cut from the GNP/PU painted PET sheets; for each sample, two copper wires were then glued with conducting epoxy to two silver paint electrodes (0.5 cm × 3 cm) previously deposited on the opposite extremities of the film’s surface (Figure 3b).

### 2.5. Piezoresistive Characterization of a Single GNP/PU Sensor

**Figure 4:**
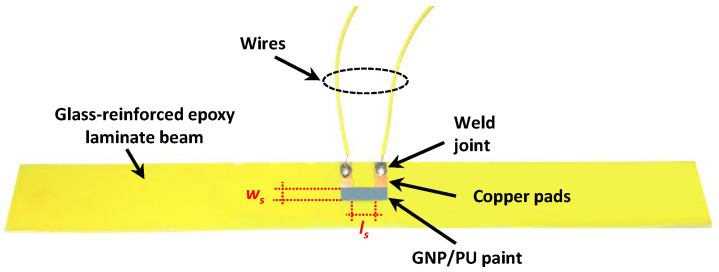
GNP/PU paint (*w_s_* = 5 mm; *l_s_* = 8 mm) deposited between two copper pads on a FR4 beam for electromechanical tests.

Polymer matrix composites loaded with conducting nanoparticles such as GNPs have already demonstrated piezoresistive behavior when subjected to mechanical stresses [3,4,6]. In fact, the electrical resistance measured between two points of the material can change when a strain is applied due to the variation in the distance/contacts between neighboring fillers forming the conductive pathways of the percolation network. Since the piezoresistive response depends not only on the filler’s concentration but also on other aspects such as, for example, the viscoelastic properties of the matrix and the interactions at the interface of the polymer and nanoparticles, electromechanical tests are generally required in order to assess the performance of the material as a strain sensor. For these purposes, a rectangular GNP/PU film (with a GNP content of 3.5 wt%) was spray-deposited on an epoxy–glass laminated beam (230 mm × 25 mm × 1.5 mm) through an airbrush mounted on a CNC x-y plotter. The deposition area, delimited with masking tape, was 16 mm × 5 mm. As shown in Figure 4, two copper pads (15 mm × 4 mm), previously patterned by photolithography and subtractive wet etching, were partially covered by the paint. The initial resistance *R*_0_ = 103 kΩ of the film was then measured by connecting the Keithely source-measurement units to the wires which were soldered to the copper electrodes. Next, similar to a common strain gauge, the variation in the resistance of the film was monitored by applying deflection to the beam. In particular, a universal testing machine equipped with a 500 N load cell, a 3-point bending fixture and an extensometer were used to perform a quasi-static monotonic flexural test. The flexure test was performed at a constant crosshead speed of 1 mm/min until a strain (ε) of 1% was reached. The variation in the electrical resistance was monitored with a Keithely 6221 current source and a Keithely 2182A nanovoltmeter.

### 2.6. Piezoresistive Charcterization of a 1D GNP/PU Sensor Array

**Figure 5:**
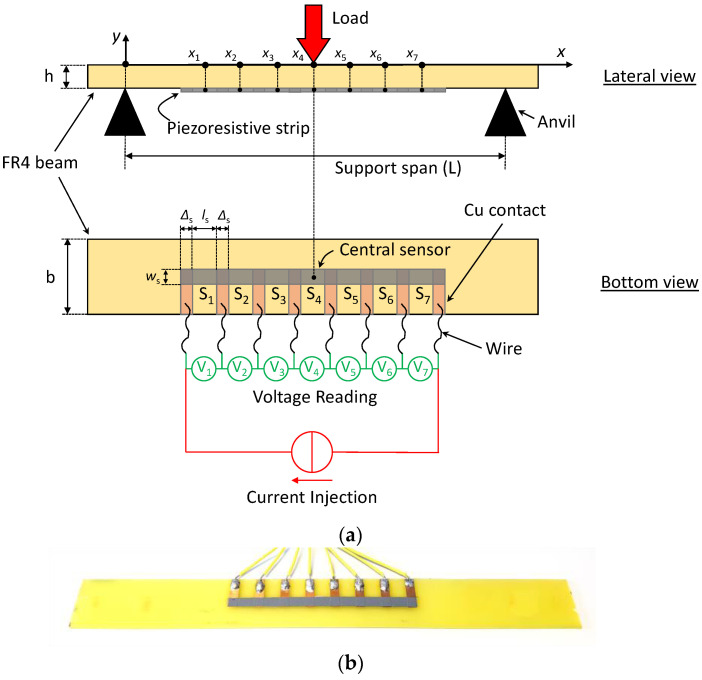
Sketch of the FR4 beam with a 1D array of seven GNP/PU painted sensors for three-point flexural tests (**a**); picture of the sample (**b**).

As shown in the sketch in Figure 5a and in the corresponding picture in Figure 5b, a rectangular stripe of piezoresistive GNP/PU paint was spray-deposited selectively at the center of an FR4 beam with dimensions of 230 mm in length, 25 mm in width (*b*) and 1.5 mm in thickness (*h*). The coating, with a width *w*_s_ = 5 mm and a length of 88 mm, partially covered 8 equidistant copper pads 15 mm in length and 4 mm in width (Δ*_s_*), previously patterned through a photolithographic process. Therefore, this resulted in the length of the coated area (gauge length) between two adjacent pads being *l*_s_ = 8 mm, and 7 in-line GNP/PU paint-based sensors (indicated as *S*_1_, *S*_2_...*S*_7_ in Figure 5b) were obtained.

As for the single sensor, the FR4 beam with GNP/PU strip was subjected to a quasi-static monotonic flexural test using a 3-point bending fixture. The distance between the supports (span) was *L* = 122 mm. The load was applied continuously at the middle of the beam at a crosshead speed of *v* = 2.5 mm/min for ~200 s in order to reach the maximum flexural strain of 1%, measured at the beam’s midpoint with a static axial clip-on extensometer. Simultaneously, the relative resistance variation as a function of time of the seven sensors was obtained with a multiple-channel data acquisition system (National Instruments USB-6210 16-channel DAQ) recording the voltage drops across the sensors in series caused by the flow of the DC current, injected with a Keithley 6221 unit between the two outer electrodes of the external sensors *S*_1_ and *S*_7_ in Figure 5b.

### 2.7. Piezoresistive Characterization of the GNP/PU Coated Surface for ERT

Electrical tomography (ET) has been used for years, especially in geophysical and medical fields. It is an investigation technique based on a non-invasive analysis of a system which uses a set of measurement at its boundaries to estimate the internal distribution of its electrical properties [15]. Basically, in order to achieve that, several electrodes are placed on the periphery of the sample being tested. Firstly, a current is injected between two electrodes and the voltages are measured between electrode pairs, then another two electrodes are selected for current injection and the voltages are recorded again, and so on. The injection–measurement pattern can follow different strategies. In particular, we adopted a modified version of the pseudo-polar injection method [25,26]. In order to reconstruct the internal changes on the basis of voltage measurements, the “inverse problem” had to be solved. Estimation of the internal distribution of the system’s properties requires the utilization of experimental data and specialized mathematical techniques available, for example, in EIDORS (Electrical Impedance Tomography and Diffuse Optical Tomography Reconstruction Software), the open-source software toolbox used in this work [27]. In addition, for the reconstruction of an ill-conditioned problem, a regularization, controlled by a hyperparameter, was adopted to calculate a stable and accurate solution. In the literature, several strategies of estimating this parameter have been presented: in this work, the most well-known method of using the L-curve was adopted [28].

**Figure 6:**
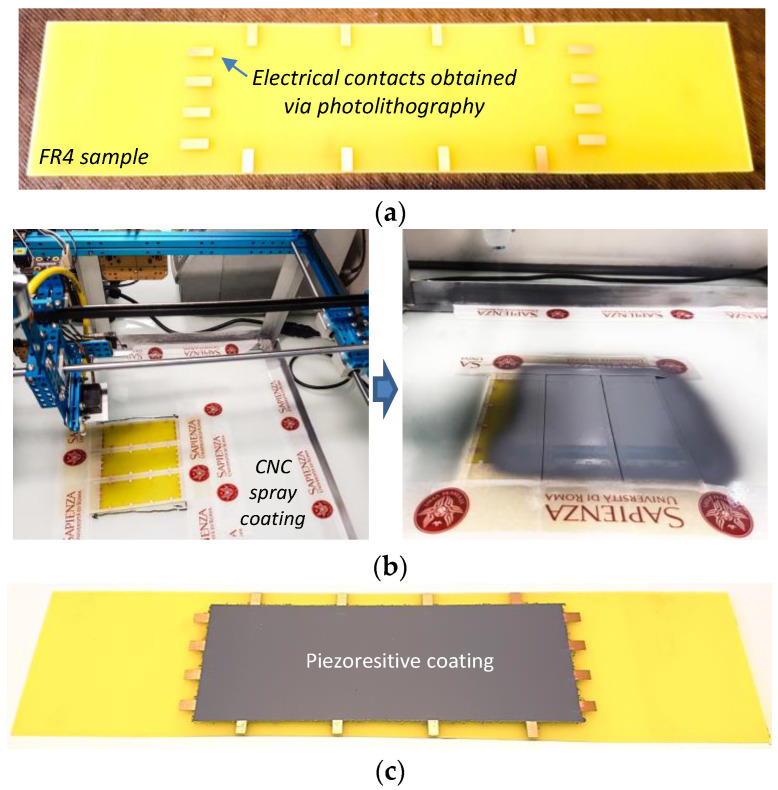
Picture of an FR4 rectangular sample with copper pads before the coating process (**a**); CNC x-y plotter equipped with an airbrush during the spray deposition of GNP-filled paint (**b**); FR4 sample with the piezoresistive coating (**c**).

The specimens for ERT were realized by depositing the piezoresistive paint on FR4 substrates, each with 16 copper electrodes realized via a photolithographic approach. In particular, two different geometries were used for the substrates: square samples of 100 mm × 100 mm × 1.5 mm and rectangular plates with dimensions of 240 mm × 50 mm × 1.5 mm. The substrates of the first type were sprayed in the center in a square area of 90 mm × 90 mm and then used to validate the ERT system for static tests, demonstrating object localization and damage recognition capabilities (Section 3.6). The others were coated on a central rectangular area of 120 mm × 40 mm and used to perform electromechanical bending tests and, hence, to demonstrate the feasibility of exploiting the piezoresistive characteristics of the nanostructured coating to extract 2D maps of the strain via ERT. In both cases, the deposition was performed with the aid of the CNC x-y system, modified to be used with an airbrush, as was used for the sample preparation described in Section 2.5 and Section 2.6. The details regarding the realization of the rectangular shaped samples are illustrated in Figure 6.

**Figure 7:**
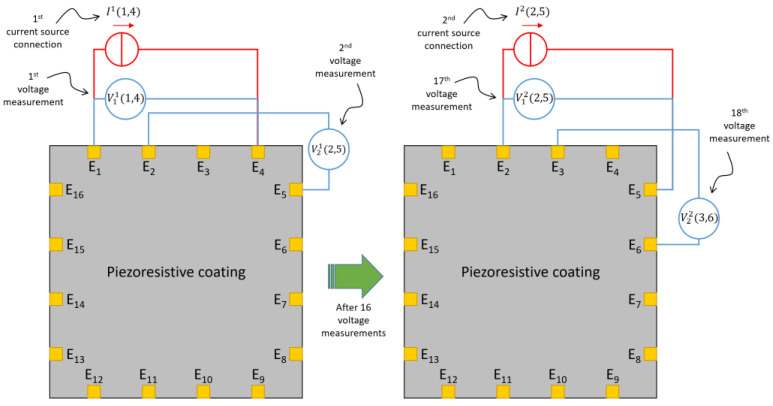
Sketches showing the adopted current injection–voltage measurement method. Only the current injection between two pairs of electrodes and two consecutive voltage measurements for each current source connection are represented for the sake of simplicity.

The acquisition was performed using a modified version of the pseudo-polar injection method, based on injecting the current $I^{n}\left(n,n+3\right)$ during the *n*-th connection of the current source between one electrode (E*_n_*) and the next (E*_n+3_*), bypassing two adjacent electrodes, as illustrated in Figure 7. The voltage measured between the *i*-th electrode and the *j*^th^ electrode is identified as $V_{m}^{n}\;\left(i,j\right)$ with:

(1) $$i=\{\begin{array}{cc} n+m-1, & \left(n+m\right)\leq 17\\ n+m-17, & \left(n+m\right)>17 \end{array}\;\mathrm{and}\;j=\{\begin{array}{cc} n+m+2, & \left(n+m\right)\leq 14\\ n+m-14, & \left(n+m\right)>14 \end{array}\;,$$

where n=1…16 is the *n*-th connection of the current source, and *m* = 1…16 is the *m*-th voltage measurement using the same current injection electrodes. Since we used 16 electrodes, 256 was the number of voltage acquisitions. Notice that, initially, a zero condition was stored (this configuration was used as a reference for tomography reconstruction), then 512 is the total number of measurements performed for each sample and used as inputs for EIDORS.

**Figure 8:**
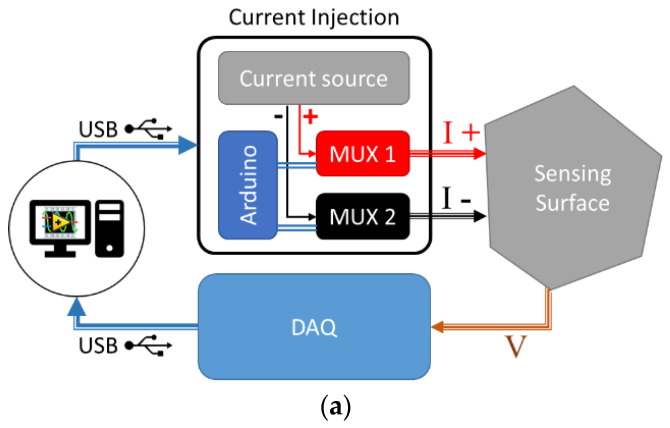

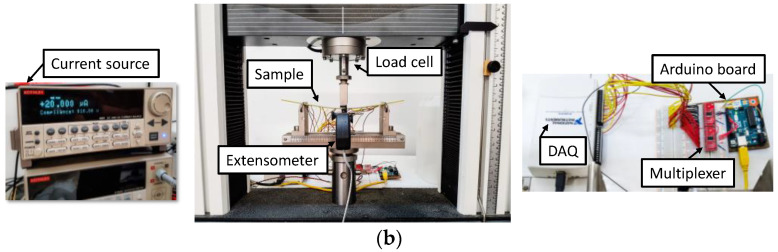
(**a**) Sketch of the ERT acquisition system; (**b**) instruments used for strain mapping.

The entire acquisition system of the measurements for ERT, schematized in Figure 8a and illustrated by pictures in Figure 8b, comprised a Keithley current source, two 16-channel analog/digital multiplexers/demultiplexers, an Arduino board and a National Instruments USB-6210 16-channel DAQ. The injection current was switched among the different electrode pairs by the two multiplexers, driven by the Arduino board. The voltage between the electrodes was measured with the DAQ board in a single-ended configuration. The whole instrumentation was controlled by a LabVIEW VI, built with a “simple state machine” architecture. In Figure 8b, the universal testing machine is also shown.

## 3. Results and Discussion

### 3.1. Morphological Charaterizaztion

**Figure 9:**
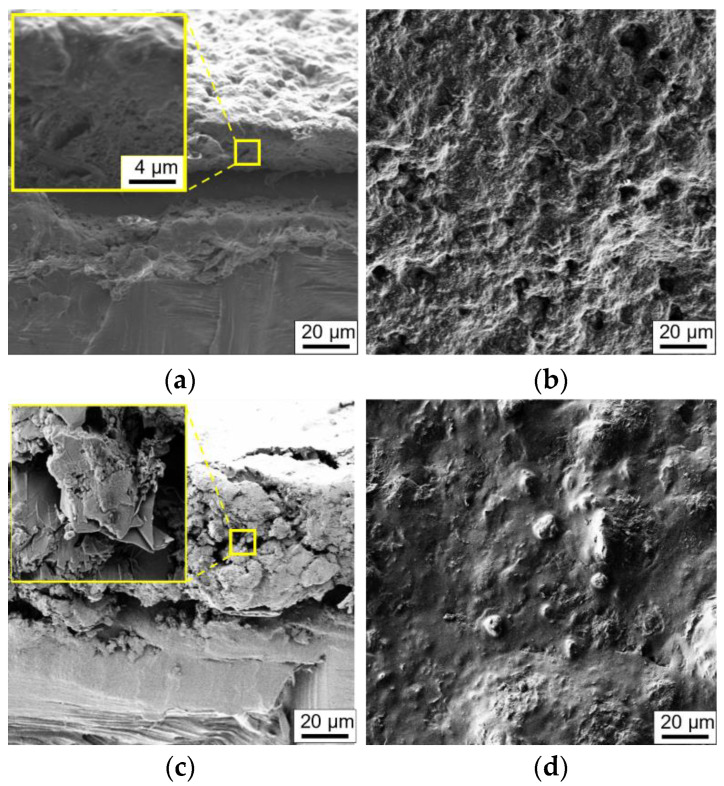
SEM images of (**a**) the cross-section and (**b**) the surface of a neat PU film, and (**c**) the cross-section and (**d**) the surface of a PU film with 3.5 wt% of GNPs.

In Figure 9a,b, we report the SEM micrographs of the neat PU paint. In Figure 9c, we show a picture of the edge fracture of the cross-section of the nanocomposite film loaded with 3.5wt% of GNPs. The coating exhibits a quite constant thickness with a value in the range of ~20 µm for the neat PU paint and of ~75 µm for the nanocomposite film. It can also be noted that the GNPs, characterized by a lateral dimension of a few micrometers, are perfectly integrated and homogenously dispersed into the polymeric matrix. The surface in Figure 9d is compact, free of voids and rather smooth.

### 3.2. Adhesion Test Results

**Figure 10:**
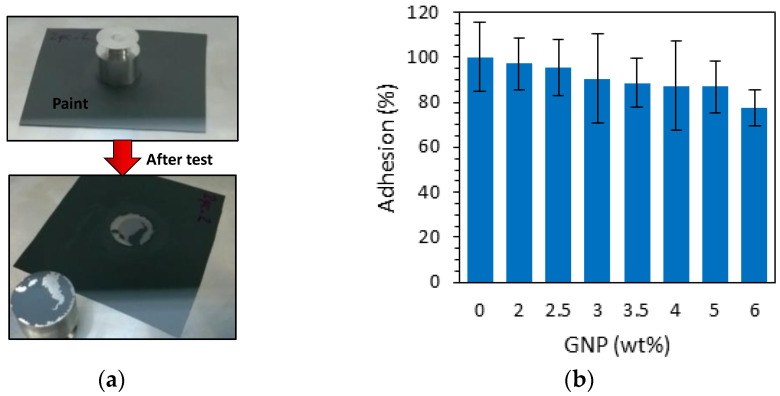
Adhesion strength between the coating and the substrate as a function of the GNP concentration: (**a**) picture of a sample before and after the test; (**b**) adhesion as a function of GNP content in the paint.

The percentage of adhesion failure stress of the coatings with different weight percentages of GNPs deposited directly on a PET substrate is reported in the bar graphs of Figure 10b. All the results are normalized with respect to the measured value for neat PU paint. It can be noticed that despite the pull-off strength tending to decrease progressively with an increase in the GNP weight fraction, it remains at ~80% of the value of neat paint even at the highest considered concentration of 6 wt%.

### 3.3. Electrical Charaterization

**Figure 11:**
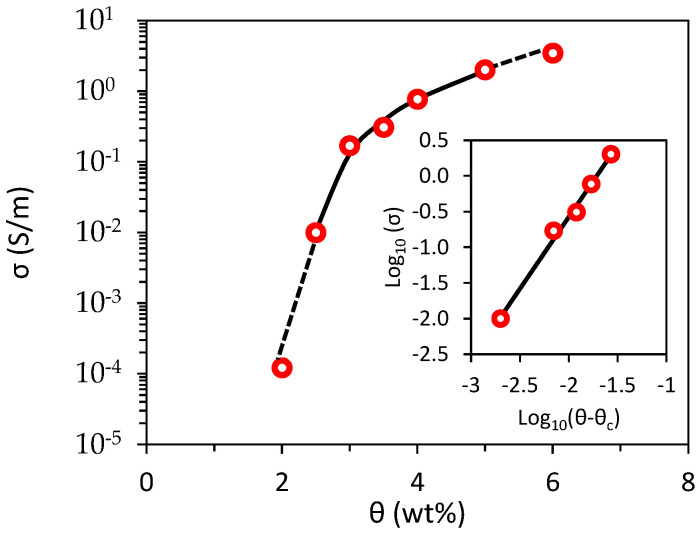
Electrical conductivity of the coatings as function of the GNP weight fraction.

The experimental data of the percolation curve are reported as symbols in Figure 11; the *σ*-*θ* curve was obtained by fitting the data with the well-known power law equation:

(2) $$\sigma \;\alpha \;{\left(\theta -\theta_{c}\right)}^{t}\;\mathrm{for}\;\theta >\theta_{c}$$

Resulting in a percolation threshold of *θ_c_* = 2.30 wt% and a critical exponent of *t* = 2.01. Notice that, due to the different polymeric matrix and process steps, the obtained percolation threshold is nearly one order of magnitude higher than that reported in [3] for a polyvinyl ester resin loaded with the same type of filler. 

Moreover, the knee point of the curve occurs approximatively at a concentration of 3 wt% of GNP instead of 0.5 wt%. Consequently, polymeric coatings with GNP concentration of 3.5 wt% were selected for the sensor applications to limit the electrical resistance to measurable values of a few tens/hundreds of kΩ.

### 3.4. Piezoresitive Response of a Single GNP/PU Sensor

**Figure 12:**
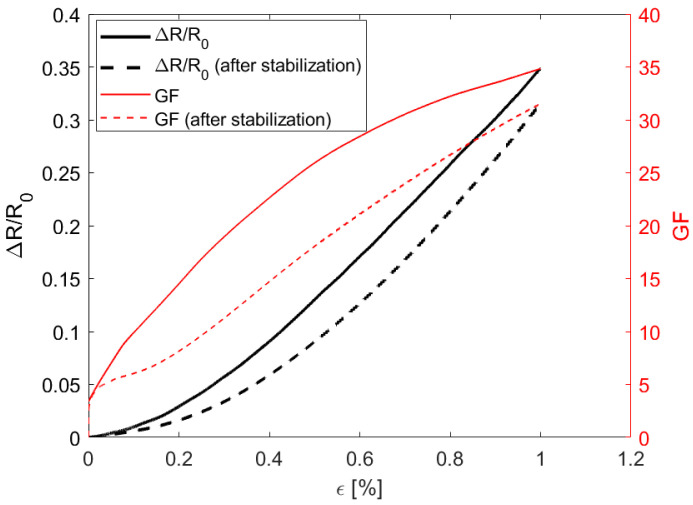
Variation in the electrical resistance (black lines) and GF (red lines) as a function of *ε*.

The electromechanical response of the GNP/PU paint-based strain sensor is reported in Figure 12. In order to quantify the performance of the produced sensors, we calculated the gauge factor (GF), defined as the ratio between the relative resistance variation and the flexural strain *ε*:

(3) $$GF=\left(\Delta R⁄R_{0}\right)/\varepsilon$$

where ∆*R* = *R*(*ε*) − *R*_0_ and *R*_0_ is the electrical resistance at resting condition. As it is well-known that piezoresistive polymeric sensors require mechanical stabilization [3,29,30], the relative variation in the electrical resistance and GF as function of ε are reported in Figure 12 after subjecting the sample to 50 cyclic flexure loading tests. We noticed that the GF after stabilization was slightly lower than that after the first test. On the one hand, it depends on a higher value of the initial resistance; on the other hand, it depends on the reorganization of a conductive sensing network constituted by the nanofillers dispersed in the polymeric matrix. It can be also noted that the minimum detectable strain is ~0.03% and the maximum sensitivity is ~33 for a strain of 1%, a significantly high value for nanocomposite polymeric strain sensors [31].

**Figure 13:**
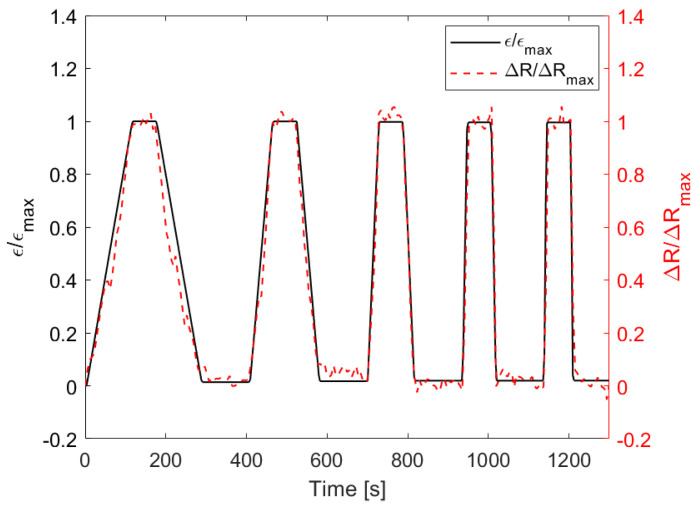
Comparison between the profile during a displacement controlled cycled bending test and the measured ∆*R* normalized with respect to its maximum value.

Finally, to verify the quality of the sensor response, we applied the deformation profile shown in Figure 13: it represents a displacement controlled cycled bending test constituted by five loading/unloading phases conducted at five different velocities, increased from 5 mm/min to 40 mm/min. When the maximum strain *ε*_max_ was reached during the loading phase, it was followed by a period of 60 s with a constant strain. Subsequently, the unloading step began: once the null strain had been reached, the machine again waited 100 s before restarting the loading phase at a higher speed. As can be seen from the figure, the variation in resistance normalized with respect to its maximum value ∆*R*_max_, corresponding to reaching *ε*_max_, accurately follows the *ε*/*ε*_max_ profile.

### 3.5. Piezoresistive Response of a Linear Sensor Array

According to Euler–Bernoulli’s theory, the maximum flexural stress (*σ_f_*) and strain (*ε_f_*) of a thin beam with a rectangular cross-section subjected to a three-point bending test are given by the well-known expressions:

(4) $$\sigma_{f}=\frac{3FL}{2bh^{2}}\;\;;\;\;\;\;\;\varepsilon_{f}=\frac{6h\delta }{L^{2}}\;\;$$

where *F* is the concentrated force applied to the middle of the beam, *L* is the span between two supports that are equally distant from the load application point, *δ* is the deflection at mid-span, and *b* and *h* are the width and thickness of the beam, respectively. In particular, Equation (4) was applied at a point at the center of either the top or bottom surfaces of the beam (and approximately along all its width when *h* << *b*). Considering Figure 5b, the profile of the strain along the *x* direction over a half-beam (the other half being symmetrical) and at the generic instant *t* is given by:

(5) $$\varepsilon (x,t)=\frac{2x}{L}\varepsilon (x_{4},t)\;\;\;\;\;\mathrm{with}\;\;\;0\leq x\leq x_{4}=\frac{L}{2}$$

where:

(6) $$\varepsilon (x_{4},t)\;=\varepsilon_{4}(t)=\frac{6h}{L^{2}}vt$$

is the time-dependent flexural strain at *x* = *x*_4_ (the center of the beam) when the deflection *δ* at mid-span increases linearly with a speed of *v* = *δ*/*t* due to the applied time-varying load.

Let us now assume the presence of seven thin sensors deposited on the bottom surface of the beam, as sketched in Figure 5a. The x-position of the centers of the first three sensors (*S*_1_, *S*_2_, *S*_3_) can be calculated as:

(7) $$x_{i}=\frac{L}{2}-\left(4-i\right)\left(\Delta_{s}+l_{s}\right)\;\;\;\;\;\mathrm{with}\;\;\;i=1...3$$

where *l*_s_ is the gauge length and Δ_s_ is the width of the copper contacts (i.e., the distance between two adjacent sensors). Hence, according to Equations (5) and (7), the strain at the midpoint of each sensing element can be expressed as:

(8) $$\varepsilon (x_{i},t)=\varepsilon_{i}(t)=\frac{2x_{i}}{L}\varepsilon_{4}(t)=\;\left[1-\frac{2\left(4-i\right)\left(\Delta_{s}+l_{s}\right)}{L}\right]\varepsilon_{4}(t)$$

The overall elongation of *S*_1_, *S*_2_ and *S*_3_ can be obtained by:

(9a) $$\Delta l_{i}(t)=\;\int_{x_{i}-l_{s}/2}^{x_{i}+l_{s}/2}\varepsilon \left(x,t\right)dx=\;\varepsilon_{i}\left(t\right)\;l_{s}\;$$

It is also noticed that the strain at the midpoint of the central sensor *S*_4_ is given by Equation (6) and its overall elongation is:

(9b) $$\Delta l_{4}(t)=\;2\int_{x_{4}-l_{s}/2}^{x_{4}}\varepsilon \left(x,t\right)dx=\varepsilon_{4}\left(t\right)\;l_{s}\;\left(1-\frac{l_{s}}{2L}\right)$$

Due to symmetry, the elongation and strain of the sensors *S*_5_, *S*_6_ and *S*_7_ coincide with those of sensors *S*_3_, *S*_2_ and *S*_1_, respectively. Moreover, the relative elongation of the first three sensors is equal to the strain at the corresponding sensors’ midpoint, whereas the relative elongation of the center sensor can be approximated to the flexural strain when the sensing element length is much smaller than the support span.

According to Equations (8), (9a) and (9b), the piezoresistive sensitivity of the *i*-th sensor can be defined with respect either to the strain at its midpoint (*GF_εi_*) or to its overall elongation (*GF_ei_*):

(10a) $$GF_{\varepsilon i}=\;\;\;\frac{\Delta R_{i}}{R_{0i}\varepsilon_{i}}=\frac{\Delta R_{i}}{R_{0i}}{\left(\frac{\Delta l_{i}}{l_{s}}\right)}^{-1}=GF_{ei}\;\;\;\;\mathrm{with}\;\;\;i=1...7\;,\;\;i\neq 4\;$$

(10b) $$GF_{\varepsilon 4}=\;\;\;\;\frac{\Delta R_{4}}{R_{04}\varepsilon_{4}}=\frac{\Delta R_{4}}{R_{04}}{\left(\frac{\Delta l_{4}}{l_{s}}\right)}^{-1}\left(1-\frac{l_{s}}{2L}\right)=GF_{e4}\left(1-\frac{l_{s}}{2L}\right)$$

where Δ*R_i_*/*R*_0*i*_ has the same meaning as in Equation (3) for the *i*-th sensor. Notice that the two definitions coincide for all the sensors except for the one at the center of the beam. For a homogeneous nanomaterial, it is possible to assume that:

(11) $$GF_{ei}=GF_{e4}=\;GF_{e}\;\;\;\;\;\;\;\;\;\;\mathrm{with}\;\;\;i=1...7\;,\;\;i\neq 4$$

In addition, at *l_s_* << *L*, it yields:

(12a) $$GF_{\varepsilon 4}≃GF_{\varepsilon i}=\;GF_{\varepsilon }\;\;\;\;\;\;\;\mathrm{with}\;\;\;i=1...7\;,\;\;i\neq 4$$

and for all the seven sensors, it yields:

(12b) $$GF_{\varepsilon }≃\;GF_{e}$$

This suggests that it is possible to define only one sensitivity curve to characterize all the sensors of the array as a function of the strain at the sensors’ midpoint. Conversely, by knowing the inverse of the sensitivity curve, it is also possible to find the local strain of the deformed beam in correspondence with the sensors, starting from the resistance measurements. 

**Figure 14:**
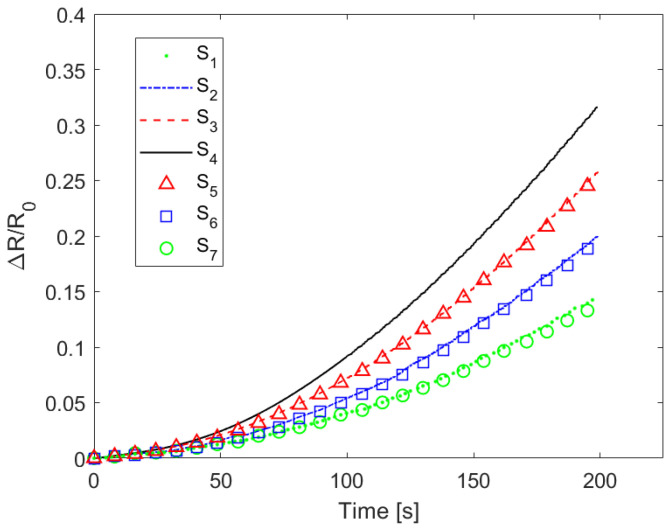
Variation in the electrical resistance measured at the contacts of sensors during the three-point mechanical bending test.

Let us consider, for example, Figure 14, in which we report the relative variation in resistance of the seven sensors as function of time when the beam shown in Figure 5b was subjected to a three-point flexural test. As expected, the sensors closest to the anvils showed lower resistance variation because they were subjected to lower strains. Moreover, the sensors equidistant from *S*_4_ showed almost the same response, thus also demonstrating the quality of the manufacturing process developed.

We then calculated the strain vs. time for the points *x_i_* (with *i* = 1...4) of the beam, corresponding to the centers of the sensors *S*_1_, *S*_2_, *S*_3_ and *S*_4_, using the measured values of Figure 14 and the *GF_ε_* of Figure 12 with:

(13) $$\varepsilon_{i}=\;\;\;\frac{\Delta R_{i}}{R_{0i}}\frac{1}{GF_{\varepsilon }}\;\;\;\;\mathrm{with}\;\;\;i=1...7\;$$

**Figure 15:**
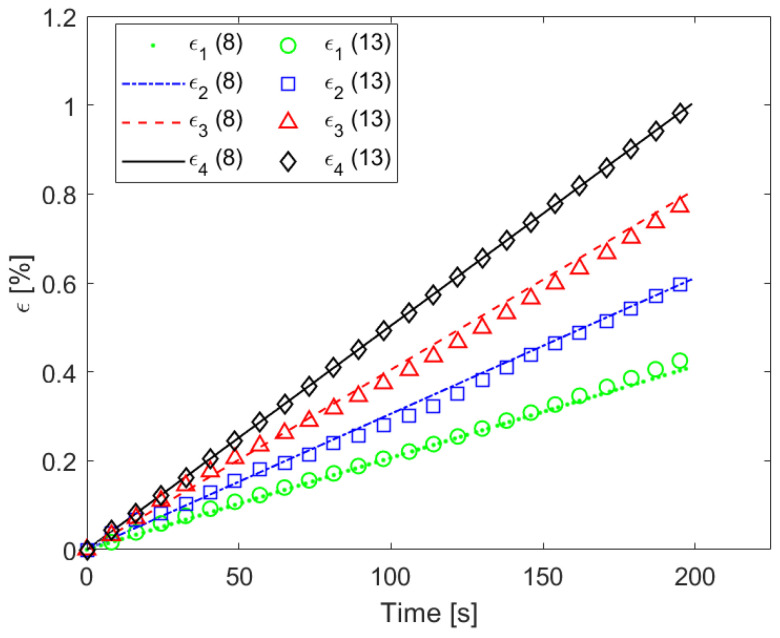
Strain vs. time at four different points (*x*_1_, *x*_2_, *x*_3_, *x*_4_) of Figure 5 during the three-point bending test obtained either by using Equation (8) (that is, knowing the position of the sensors, the beam geometry and the crosshead deflection vs. time) or by using Equation (13) (that is, from the measurements of the variation in resistance of the sensors and the piezoresistive sensitivity).

The extracted values are reported in Figure 15, in which the strain profiles obtained directly by Equation (8) are also shown to demonstrate the good agreement between the strains calculated from the deflection at mid-span and those obtained by the resistance measurements and sensitivity.

### 3.6. Charaterization of the Sensors’ Surface

The 100 mm × 100 mm × 1.5 mm square-shaped samples described in Section 2.7 were firstly used to experimentally check the ERT setup by investigating the feasibility of localizing an electrically conducting object placed on the piezoresistive film.

**Figure 16:**
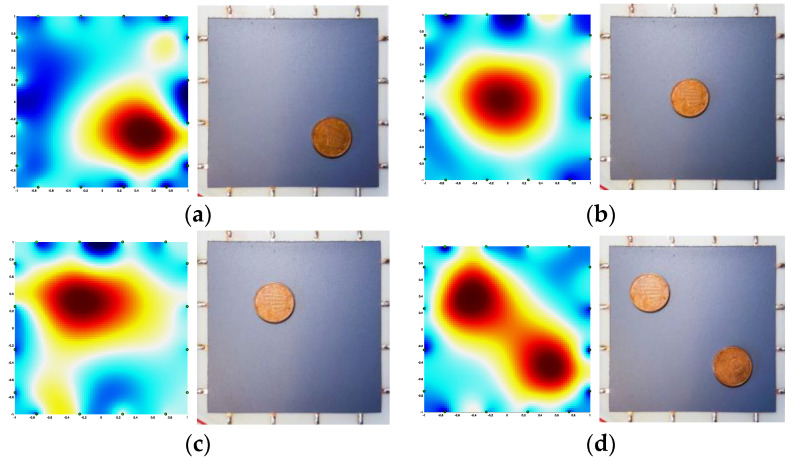
ERT object localization tests: (**a**–**c**) single coin in different positions; (**d**) two coins on the surface.

**Figure 17:**
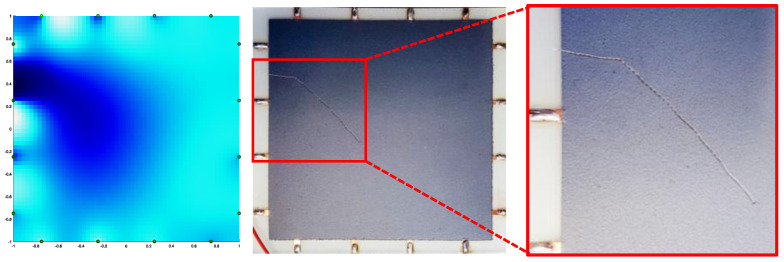
ERT damage identification test.

After recording the reference configuration (no load applied and no objects on the sample surface) a 5-cent coin was placed and moved over the painted sample in different positions. As the electrical conductivity of the copper-covered steel coin was much greater than that of the paint with 3.5 wt% of GNPs reported in Figure 11, it modified the current path injected by the electrodes at the sample’s edges. Therefore, as shown in Figure 16a–c, the algorithm interpretated that as a local variation of the effective electrical conductivity and consequently provided a 2D colored map disclosing the presence of the object. After we laid a second coin on the surface, the ERT test also located the two separate objects correctly (Figure 16d). Furthermore, after scratching the paint surface with an indenter, a damage recognition test was performed (Figure 17). As shown by the reconstructed image in Figure 17, the area around the scratch is of a deeper blue color, as the damage locally decreased the effective conductivity of the coating.

**Figure 18:**
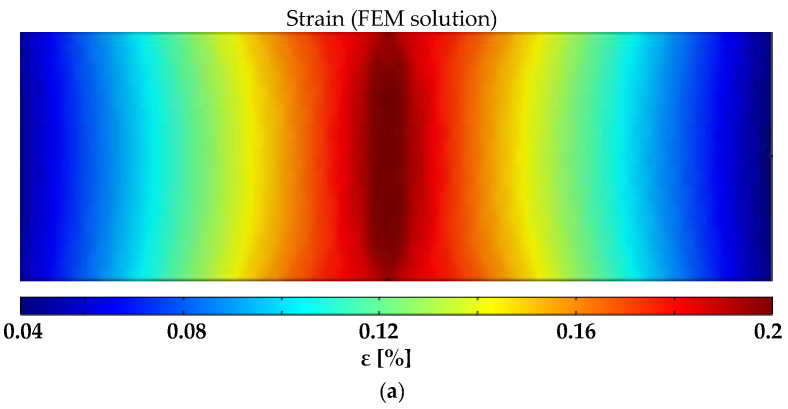

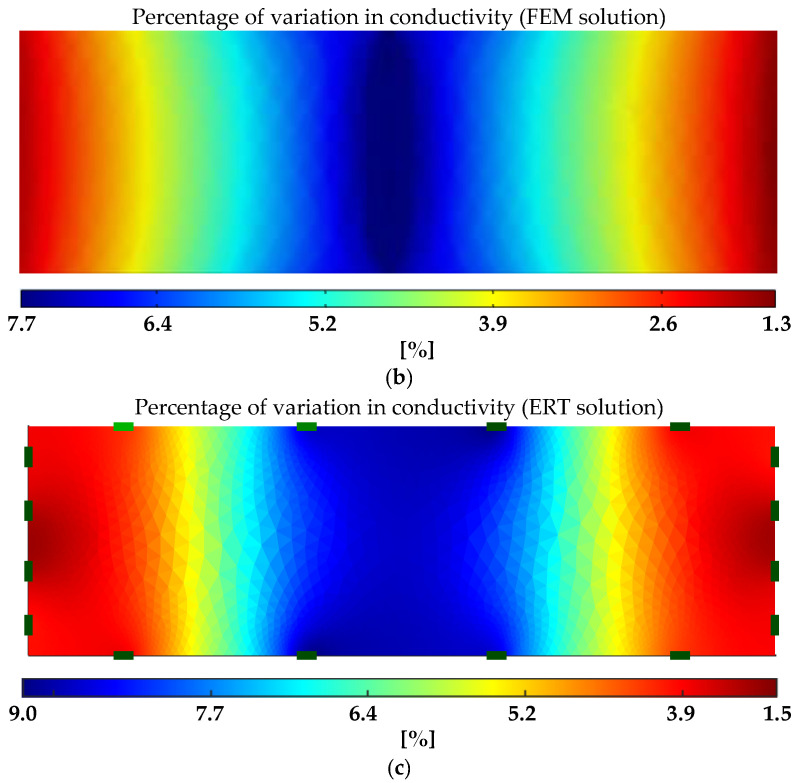
FR4 sample with the piezoresistive coating shown in Figure 6 subjected to a flexural test: (**a**) strain map and (**b**) corresponding map of the percentage of variation in conductivity simulated by COMSOL; (**c**) map of the percentage of variation in conductivity reconstructed from the ERT measurements.

Finally, the system was used to monitor the state of deformation of the rectangular FR4 plate described in Section 2.7 subjected to a 3-point bending test ((Figure 8b) with a span of 160 mm and reaching a maximum flexural strain of 0.2%. COMSOL Multiphysics software was also used for finite element method (FEM) analyses and for predicting the electromechanical response. The dimensions of the substrate, of the coating and of the electrodes, as well as the effective conductivity at rest of the GNP-based paint and its piezoresistive coefficients vs. strain derived from the GF curve in Figure 12, were all inputs for the FEM code. The percentage of variation in the conductivity map obtained by EIDORS and evaluated using the voltages measured at the different electrodes when the maximum flexural strain was applied is reported in Figure 18c. It can be observed that the reconstructed change in the conductivity pattern has a color distribution close to the one obtained with the FEM simulation (Figure 18b) and is compatible with the simulated strain map of the mechanically loaded plate (Figure 18a).

## 4. Conclusions

In this work, the development of a polyurethane GNP-loaded piezoresistive paint has been presented with the intent of exploring its potential use for discrete strain and spatial sensing.

Morphological characterization through SEM imaging showed that the nanostructures were dispersed homogeneously within the polymeric matrix and that the average film thickness was 70–80 µm. Electrical tests provided information about the DC conductivity of the composite and its variability as a function of the fraction of fillers in the PU paint, thus indicating the GNP concentration (3.5 wt%) to be adopted for the realization of sensitive piezoresistive sensors and the value of ~0.35 S/m for the FEM simulations. Moreover, adhesion tests verified that the presence of the nanostructures inside the paint film lowered the adhesion strength by only 20% with respect to neat paint.

In order to investigate piezoresistive properties of the modified PU paint, several kinds of strain sensors have been produced. A significant performance was demonstrated for the GNP/PU sensor, showing a minimum local detectable strain of ~0.03% and a gauge factor of ~33 at 1% of strain. Moreover, through the monitoring of the variation in the resistance of the sensors constituting a linear array on an FR4 beam, it was possible to extract, in a discrete way, the strain vs time at seven points of the substrate during loading. Finally, the feasibility of localizing objects and damage and recognizing deformations on surface coated with the piezoresistive paint by using the ERT technique was verified. We believe that these capabilities can be further improved by using machine learning algorithms, and future developments will be addressed to low-velocity impact detection, a particularly challenging objective, especially in the aeronautical field.

## Data Availability

Not applicable.
